# Chaos-based block permutation and dynamic sequence multiplexing for video encryption

**DOI:** 10.1038/s41598-023-41082-9

**Published:** 2023-09-07

**Authors:** Heping Wen, Yiting Lin, Zhiyu Xie, Tengyu Liu

**Affiliations:** 1 School of Electronic Information, University of Electronic Science and Technology of China, Zhongshan Institute, Zhongshan, 528402 China; 2 School of Information and Communication Engineering, University of Electronic Science and Technology of China, Chengdu, 611731 China

**Keywords:** Mathematics and computing, Physics

## Abstract

This paper proposes a video security transmission enhancement algorithm based on block permutation and dynamic multiplexing sequences encryption based on 4D autonomous hyperchaotic system. Firstly, we employ the block permutation encryption and diffusion confusion encryption module, which is based on dynamic multiplexing chaotic sequences, to encrypt the plaintext and obtain the ciphertext. Subsequently, the hash value of this round’s ciphertext is utilized to generate the chaotic key, produced by the multiplexing sequence of this round after mathematical processing. Then, the key is used to generate the chaotic sequence to confuse the N-th of the multiplexed sequence, and the next round of multiplexed sequence is obtained. If the current round of chaotic sequence has been completely confused, the chaotic sequence is re-generated to generate a new multiplex sequence by using the key generated by the current round key and the initial key. Finally, the above steps are repeated for the encryption of each frame of the video. Compared with the traditional permutation coding algorithm, it increases the difficulty of estimation or recognition while ensuring efficiency, and effectively improves the avalanche effect of the algorithm. Through frame by frame ciphertext closed-loop feedback, it has the ability to resist known plaintext attack and selected plaintext attack. The results show that the scheme has high security and significant diffusion characteristics, and can effectively resist various common cryptographic attacks.

## Introduction

In recent years, secure transmission and data protection of multimedia data has become increasingly important. With the rapid development of information technologies such as the Internet of Things, big data, and cloud computing, the security and privacy protection issues of multimedia, especially video sharing applications, have become increasingly prominent. In the absence of a secure system, video data will be vulnerable to attack. Therefore, ensuring video data security and transmission protection is crucial. Because of its high sensitivity to initial conditions and control parameters, good pseudo-randomness, ergodicity, long-term unpredictability of orbits and other aspects, chaos has many similarities with permutation, confusion and diffusion in cryptography, and has been widely used^1–5^ in image and text encryption. At the same time, more and more chaotic encryption methods have been proposed, such as biological coding^6–8^, frequency domain encryption^9–11^, quantum encryption^12–14^, bit plane encryption^15–17^, thumbnail-preserving encryption^18–21^ and so on^22–29^. However, compared with information such as images and texts, video information has the characteristics of high information redundancy, strong pixel correlation, and discrete distribution of key information, which requires encryption and transmission of large amounts of data. Most traditional chaotic encryption methods^30–32^ are not suitable for video encryption because their encryption algorithms cannot process large amounts of video data in real time. In this regard, it is urgent to propose a video encryption algorithm that is both secure and efficient.

Throughout the international research status, in view of the challenges posed by video data security, researchers have devoted themselves to a large number of video encryption algorithms in the past two decades and achieved good results^33–35^. Its algorithm^36–38^ is generally divided into two types: complete encryption and selective encryption. Both have their own advantages and disadvantages. Complete encryption is usually used for a small amount of data with strict security and confidentiality requirements. Selective encryption is suitable for the case where the amount of data is large and the transmission content needs to be guaranteed to be real-time. Therefore, according to different encryption requirements, the proposed algorithms are also different. These studies are very important for video encryption and data security. Unfortunately, with the development of society, it is impossible to meet the needs of today ’s society only from the perspective of security or efficiency. There have been more and more occasions that require both security and real-time content. From the perspective of social needs, most of the previous studies have certain limitations: (1) The use of multiple rounds of encryption, iterative encryption and other methods can effectively improve the quality of encryption, but there are problems such as low execution efficiency and information redundancy; (2) Selective encryption only important or sensitive video information is selected for encryption to reduce the computational complexity, but it does not apply to all video encryption; (3) For complex chaotic systems, generating a large number of chaotic sequences will greatly increase the encryption time.

In order to solve the above problems, this paper proposes an enhanced algorithm for secure video transmission based on block permutation and dynamic multiplexed sequence encryption of 4D autonomous hyperchaotic systems. To address the problem of time-consuming video encryption, we use dynamic multiplexing on the chaotic sequence, dividing the chaotic sequence generated in each round into N pieces, using the eigenvalues of the ciphertext image of the previous frame, and then processing it through the chaos system to generate a small sequence of N parts confused with one N parts of the chaotic sequence, and this whole chaotic sequence is used as the chaotic sequence for the next encrypted plaintext image. When N blocks of the chaotic sequence of a round have been confused one by one, the chaotic sequence required for the next round of encryption is generated using the hash of the ciphertext image of this frame processed and then the XOR operation is performed with the initial chaotic key parameters. Meanwhile, we propose a block permutation encryption algorithm based on chaotic sequences. Compared with traditional permutation and encoding algorithms, its permutation algorithm is dynamically adjusted according to chaotic sequences, so that it achieves good encryption results with as little time as used. Its subsequent confusion and substitution and ciphertext feedback enhance the cryptographic security of the algorithm and the cryptographic avalanche effect, which greatly improves the resistance to cryptographic attacks. Theory analysis and experimental results show that the scheme is highly secure and efficient, and can effectively resist various common cryptographic attacks. Therefore, the method proposed in this paper can better improve the security and reliability in the video transmission process, and is expected to propose a new way of thinking as a way to ensure secure communication in the era of big data.

The rest of this paper is organized as follows. Section Introduction succinctly describes the theory related to this algorithm. Section Relevant theories specifies our proposed chaotic encryption algorithm method. Section Design of encryption algorithm presents the experimental simulation results. The last section concludes the paper.

## Relevant theories

### 4D autonomous hyperchaotic system

The 4D autonomous hyperchaotic system^39^ used in this paper is obtained by iterating 3D system, where the state equation of the 3D system is defined as follows:

1 $$\begin{aligned} {\left\{ \begin{array}{ll} \dot{x}_{1}=a(x_2-x_1)\\ \dot{x}_{2}=cx_1-x_1x_3\\ \dot{x}_{3}=-bx_3+x_1x_2\\ \end{array}\right. } \end{aligned}$$

where $$ a=35$$, $$b=3$$, $$c=35$$, the system has three unstable equilibrium points, denoted as *O*(0, 0, 0), $$P_+ (\sqrt{105},\sqrt{105},35)$$, $$P_- (-\sqrt{105},-\sqrt{105},35).$$

**Figure 1:**
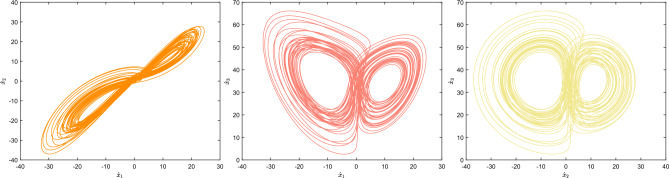
2D chaotic attractor phase diagram of 4D autonomous hyperchaotic system.

**Figure 2:**
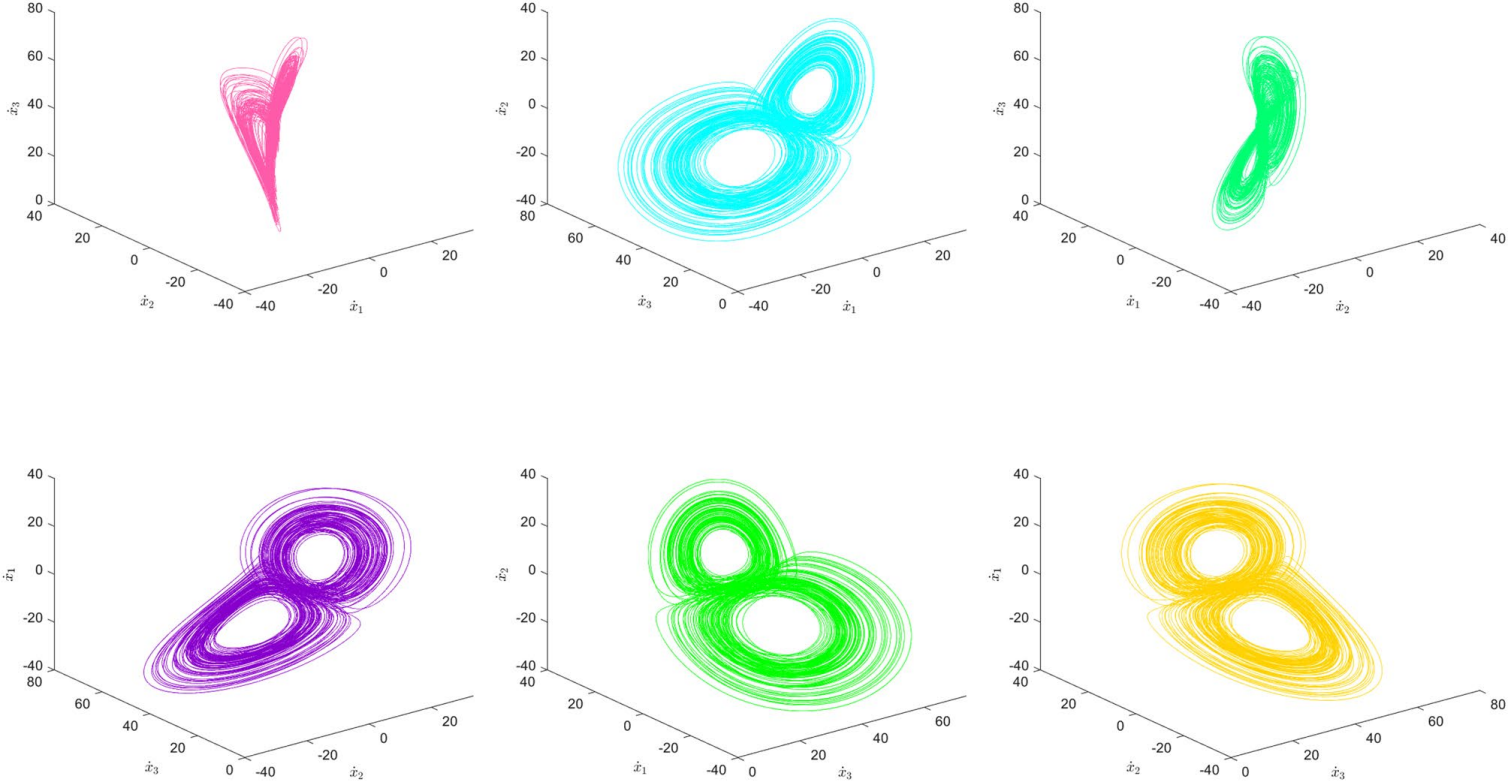
3D chaotic attractor phase diagram of 4D autonomous hyperchaotic system.

In the current system, the first three equations can be introduced into the controller $k_ 1 x_ 4,k_ 2 x_ 4,k_ 3 x_ 4$ respectively, and $\dot{x}_4=-dx_ 1$ can be added to construct the following 4D autonomous hyperchaotic system:

2 $$\begin{aligned} {\left\{ \begin{array}{ll} \dot{x}_{1}=a(x_2-x_1)+k_ 1 x_ 4\\ \dot{x}_{2}=cx_1-x_1x_3+k_ 2 x_ 4\\ \dot{x}_{3}=-bx_3+x_1x_2+k_ 3 x_ 4\\ \dot{x}_{4}=-dx_1\\ \end{array}\right. } \end{aligned}$$

where the chaotic parameters are $$ a=35$$, $$ b=3$$, $$c=35$$, $$ k_ 1=1$$, $$ k_ 2=0.2$$, $$ k_ 3=0.3$$, $$ d=5$$, the Lyapunov exponents of the system are $$ LE_ 1=0.5$$, $$LE_ 2=0.2117$$, $$LE_ 3=0$$, $$LE_ 4=-38.7068$$, and the system exhibits hyperchaotic behavior. At the same time, there are two positive Lyapunov exponents when $$ a=35$$, $$ b=3$$, $$c\in [32,70]$$, $$d\in [1,25]$$, the system is in a hyperchaotic state. The numerical simulation results of hyperchaotic attractors are shown in Figs. 1 and 2.

### Block permutation

**Figure 3:**
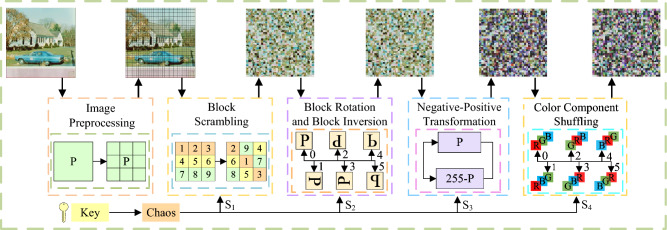
Flow chart of block permutation.

In this section, chaos sequences denoted as $S_1,\,S_2,\,S_3,\,S_4$ are formed by utilizing the key to generate chaos. Figure 3 illustrates the block permutation flowchart utilized in this research. The procedure consists of the following specific steps:

**Step 1**. Chaotic sequence generation

The plaintext image is input and the MD5 hash value is obtained using a hash function, which is then processed into a key that complies with the chaotic range. Four pseudorandom sequences are obtained through the chaos system and preprocessed according to the following method:

3 $$\begin{aligned} {\left\{ \begin{array}{ll} S_1'=\lfloor (S_1\times 10^{10})mod((H\times W)/8^2)\rfloor \\ S_2'=\lfloor (S_2\times 10^{10})mod6\rfloor \\ S_3'=\lfloor (S_3\times 10^{10})mod2\rfloor \\ S_4'=\lfloor (S_4\times 10^{10})mod6\rfloor \\ \end{array}\right. } \end{aligned}$$

where $\lfloor \cdot \rfloor$ denotes rounding towards negative infinity and *mod*($\cdot$) represents the modulo function, $H\times W$ are the size of image.

**Step 2**. Block scrambling

**Figure 4:**
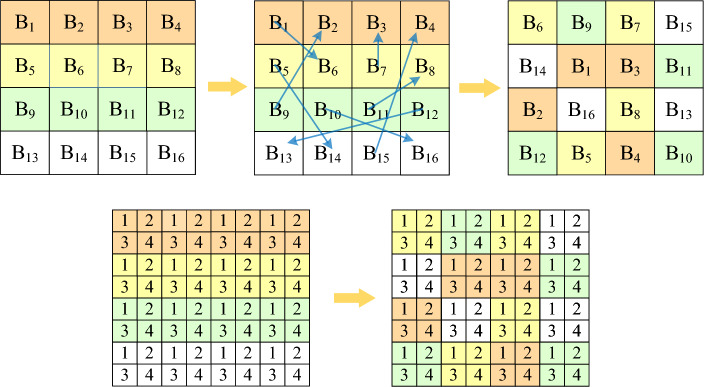
Schematic diagram of block scrambling.

The sub-blocks in the processed image $B_1$ are scrambled using the chaotic sequence $B_0$ to generate the matrix $S_1'$, as shown in Fig. 4. The scrambling method is as follows:

4 $$\begin{aligned} {\left\{ \begin{array}{ll} t=B_0(x,y,z,o)\\ B_0(x,y,z,o)=B(x,y,z,S_1'(o))\\ B_0(x,y,z,S_1'(o))=t\\ \end{array}\right. } \end{aligned}$$

where *x*, *y*, *z* represent the rows, columns and dimensions of image *B*; *Bx* is the edge length of the block and $o = 1,2,...,m\times n/(Bx \times Bx)$ which is the chunked serial number.

**Step 3**. Block rotation and block inversion

**Figure 5:**
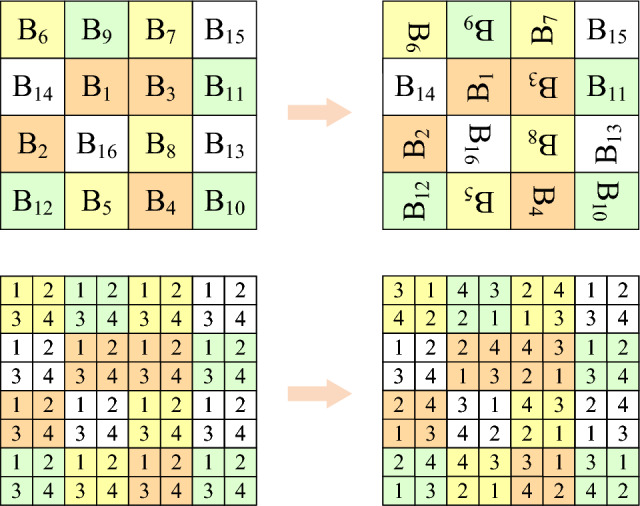
Schematic diagram of block rotation and block inversion.


The matrix $B_2$ is generated by performing block selection and reversal encryption on the data in sub-block $B_1$ using the sequence $S_2'$, as illustrated in Fig. 5.

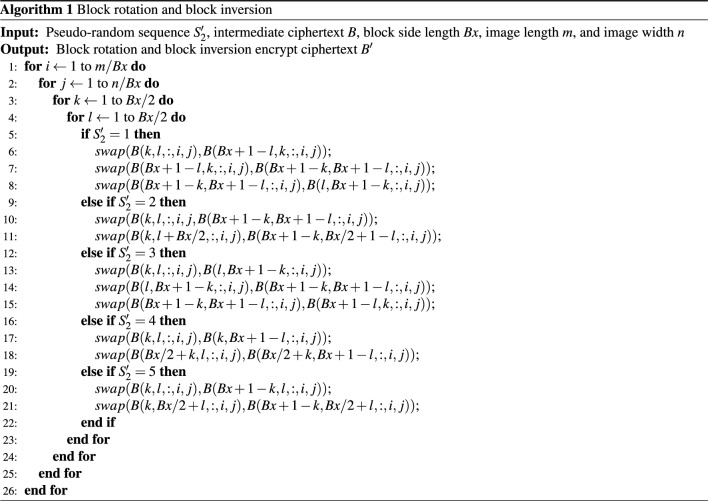


Algorithm 1 provides pseudocode for generating block rotation and block inversion. Depending on different conditions, different angles are used to rotate the matrix $B_1$. Specifically, when $S_2' \in [1,5]$, the matrix $B_1$ is rotated clockwise 90$^ \circ$, 180$^\circ$, 270$^\circ$, or flipped horizontally or vertically. In the current situation, each block achieves the purpose of permutation based on its specific conditions, providing theoretical support for block rotation.

**Step 4**. Negative-Positive transformation

When $S_3'$ = 1, certain values within matrix $B_2$ are subtracted from 255. However, when $S_3'$ = 0, no operation is conducted on matrix $B_2$. Finally, matrix $B_3$ is generated according to the following specific procedures:

5 $$\begin{aligned} {\left\{ \begin{array}{ll} B_3(x,y,z,i,j)=255-B_2(x,y,z,i,j) \quad if \quad S_3' =1\\ B_3(x,y,z,i,j)=B_2(x,y,z,i,j) \quad if \quad S_3' =0\\ \end{array}\right. } \end{aligned}$$

where *x*, *y* are the number of rows and columns of matrix $B_2$ with specific values of 1, 2, ..., *H* , 1, 2, ..., *W*; *i*, *j* are the number of rows and columns of the matrix $B_2$ after blocking, with the specific values are $1,2,..., \frac{H}{Bx}$ ; $1,2,..., \frac{W}{Bx}$; z is the dimensions of matrix $B_2$.

**Step 5**. Color component shuffling

**Figure 6:**
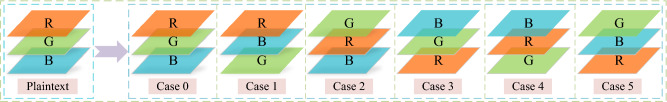
Schematic diagram of color component shuffling.


Matrix $B_4$ is generated through corresponding color component transformation on the positively and negatively transformed matrix $B_3$, based on the sequence $S_4'$. The specific processing method is shown in Fig. 6.

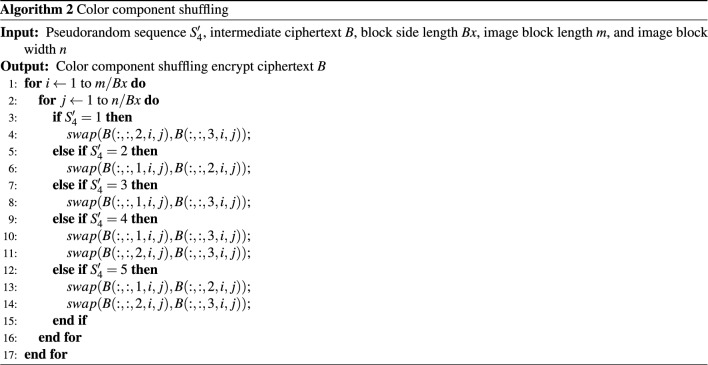


Algorithm 2 presents the pseudocode of a color component swapping algorithm between blocks, which exchanges color components of different layers through various swapping conditions, providing theoretical support for color classification scrambling.

## Design of encryption algorithm

**Figure 7:**
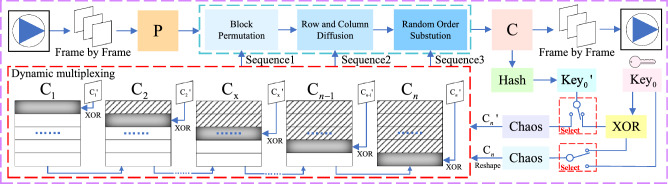
Flow chart of the proposed encryption algorithm.

To address current issues, this article proposes a video encryption scheme based on chaos-based block permutation and dynamic sequence multiplexing, which effectively improves the efficiency and security of the encryption algorithm. The proposed scheme is resistant to cryptographic attacks under the condition of plaintext-ciphertext correlation. The specific encryption algorithm is presented in Fig. 7.

### Chaotic initial value disruption and sequence pretreatment

This article utilizes the MD5 hash function to establish correlations between frames in the video, which enhances the algorithm’s resistance to brute-force attacks due to its collision resistance properties. In addition, within cryptography, the original chaotic sequence generated cannot directly serve as an encryption tool. To use the sequence in encryption, mathematical methods must be applied to ensure that each value falls within the required range for the algorithm, while retaining the chaotic characteristics. In this algorithm, three chaotic sequences *Q*, *S*, *I* are required to encrypt one frame, and specific steps for processing are presented.

**Step 1**. Reads the plain image hash

The hash MD5 algorithm is used to extract the eigenvalues of the image, a 32-bit hexadecimal number can be obtained, with each bit is represented as *h*(*x*), where $h(x)\in \left\{ 0,...,14,15 \right\}$ and $x=[1,2,3,...,32]$. $key_0$ is the initial key, which is related to the first plaintext image of the video. The specific formula is as follows:

6 $$\begin{aligned} key_0=(h(1)\oplus h(4)\oplus h(32)) mod 24 + 1 \end{aligned}$$

**Step 2**. Initial value scrambling and generating the initial parameter key

The feature values generated by the MD5 hash are used to disrupt the initial values of chaotic maps, thereby increasing their ability to resist differential attacks. The specific formula is as follows:

7 $$\begin{aligned} key_1=(h(1)\oplus h(6)\oplus h(11)\oplus h(16)\oplus h(21)\oplus h(26)\oplus h(31)+key_0 ) mod 24 + 1 \end{aligned}$$

where $\oplus$ is a bitwise XOR operation; $key_1$ is the key updated after the disturbance from MD5, which will be used to generate the chaotic sequence.

**Step 3**. Chaotic sequence preprocessing

$key_1$ is used as the chaotic parameter key, an initial chaotic sequence $S_0$ is generated. The $S_0$ sequence is the first four-dimensional sequence of each iteration of chaos. For ease of encryption operations, the specific mathematical processing is as follows:

8 $$\begin{aligned} {\left\{ \begin{array}{ll} Q = \big | \lfloor S_0 \times 2^{10}\rfloor \big | mod256 &{} (a)\\ S = \big | \lfloor S_0 \times 2^{10}\rfloor \big | mod256 &{} (b)\\ {[}\sim ,I]=sort(S_0) &{} (c)\\ \end{array}\right. } \end{aligned}$$

where $mod(\cdot )$ is a remainder function whose result is the remainder obtained by dividing two numerical expressions; $sort (\cdot )$ denotes sorting each element of the sequence from smallest to largest and *I* is the resulting sort index of $S_0$.

### The proposed algorithm

#### Encryption process

Since the video has the characteristics of multi-frame image combination, this paper proposes a multiplexing sequence algorithm based on plaintext-ciphertext association. In terms of security performance, this algorithm first uses the first frame plaintext association to generate the initial $key_0$, and then mathematically processes the hash generated by the frame-by-frame ciphertext association with the key of the previous ciphertext image to generate a new key, which greatly improves the algorithm’s resistance to cryptanalysis. In terms of efficiency, due to the introduction of confusion mechanism, each round of encryption only requires the use of a key to generate a complete initial chaotic matrix. Starting from the second frame, the initial sequence is confusing step by step to achieve reuse, achieving encryption operations for multiple frames of images, and saving time for each generation of long sequences. The multiplexed sequence ensures that the sequence used for each frame image encryption is different from other frame images by iterative obfuscation. This algorithm can improve the efficiency of video encryption while ensuring security, making it more effective and reliable. Take a video with an encryption size of $H \times W$ and *z* frames as an example, assuming that the multiplexing frequency is *n*, each round can process $n+1$ frames of video, and the encryption steps for each round are as follows:

**Step 1**. Generate multiplexed chaos matrix

First, read the first frame of each round of video images to generate the initial $key_0$. The $key_0$ will be used as the initial parameter to generate a chaotic sequence $S_0$ of length $H \times W$. Next, it will be reconstructed into a reuse chaotic matrix $R_0$ with a height *H* and width *W* that matches the size of the video.

**Step 2**. Encrypt the first frame

After generating the reuse chaotic matrix $R_0$, perform encryption on the first plaintext image *P* using the classical permutation-diffusion-substitution structure, resulting in the first ciphertext image $C_z(z=1)$. Next, taking the first frame image as an example, we will provide a detailed explanation of the specific encryption process for each frame image in this algorithm.

(1) Block permutation

In this paper, block permutation is used to process the image. In this process, four keys need to be used to generate a chaotic sequence, which is selected as follows:

9 $$\begin{aligned} {\left\{ \begin{array}{ll} a_1=R_0(1,1) \\ a_2=R_0(1,W) \\ a_3=R_0(H,1) \\ a_4=R_0(H,W) \\ \end{array}\right. } \end{aligned}$$

where $a_1,a_2,a_3,a_4$ represents the four keys used, and the chaotic generation sequences is used to generate the sequence $S_1', S_2', S_3', S_4'$ by Eq. (3), respectively for the four modules of block replacement. The specific encryption process is described in Section Block permutation, and the initial block permutation image *B* can be obtained after encryption.

(2) Row and column diffusion

The multiplexing chaotic matrix is $R_0$ transformed into a row $S_0$ vector of length $H \times W$, and then $S_0$ processed using Eq. (8a), before being restructured into a chaotic matrix *Q* with height *H* and width *W*. The plaintext image *B* is shuffled by taking the modulus of the rows and columns followed by diffusion, resulting in the diffusion ciphertext image *M*. The specific operational method is as follows:

- The block permutation image *B* is diffused into a row-diffused image *D* using the following formula:
  10 $$\begin{aligned} D_i = {\left\{ \begin{array}{ll} (B_1+B_H+\lfloor Q_1 \times 2^{10}\rfloor )mod256 &{} if \quad i=1\\ (D_{i-1}+B_i+B_{i+1} +\lfloor Q_i \times 2^{10}\rfloor )mod256 &{} if \quad i=[2,W-1]\\ (D_{H-1}+B_H+\lfloor Q_H \times 2^{10}\rfloor )mod256 &{} if \quad i=W\\ \end{array}\right. } \end{aligned}$$
  where, $D_i,B_i,Q_i$ represents the $i-th$ row sequence of the row diffusion image *D*, and chaos matrix *Q*, respectively.
- The matrix *M* is obtained by column diffusion on the basis of the matrix *D*. The specific formula is as follows:
  11 $$\begin{aligned} M_j = {\left\{ \begin{array}{ll} (D_j+D_W+\lfloor Q_1 \times 2^{10}\rfloor )mod256 &{} if \quad j=1\\ (M_{j-1}+B_j+B_{j+1} +\lfloor Q_j \times 2^{10}\rfloor )mod256 &{} if \quad j=[2,H-1]\\ (M_{W-1}+B_W+\lfloor Q_W \times 2^{10}\rfloor )mod256 &{} if \quad j=H\\ \end{array}\right. } \end{aligned}$$
  where $D_j,M_j,Q_j$ denote the i-th column sequence of the column diffusion image *D*, column diffusion image *M*, and the chaos matrix *Q*, respectively.

(3) Random order substitution

According to Eq. (8b, c), the index sequences *I* of sequences *S* and $S_0$ can be obtained, respectively, and the random order replacement operation of image *M* can be performed using *S* and *I*, the specific formula is as follows:

12 $$\begin{aligned} C_{I_{i,j},j} = {\left\{ \begin{array}{ll} (M_{I_{i,j},j}+M_{I_{H,W},W}+\lfloor 2^{32} \times S_{I_{i,j},j}\rfloor )mod256 &{} for \quad i=1,j=1\\ (M_{I_{i,j},j}+M_{I_{i-1,W},W}+\lfloor 2^{32} \times S_{I_{i,j},j}\rfloor )mod256 &{} for \quad i=[2,W],j=1\\ (M_{I_{i,j},j}+M_{I_{i,j},j-1}+\lfloor 2^{32} \times S_{I_{i,j},j}\rfloor )mod256 &{} for \quad i=[1,W],j=[2,W]\\ \end{array}\right. } \end{aligned}$$

Thus, the final ciphertext image $C_z(z=1)$ of the first frame can be obtained.

$\mathbf{Step ~3}$. Blocking for multiplexing chaotic matrix

For a multiplexed chaotic matrix of size $H\times W$, it can be arbitrarily divided into $r_i$ block which size $\frac{H}{n}\times W$ block chaos matrix $C_{I_i}$ by row. Where *i* represents the i-th block of the partitioned chaotic matrix, $i=[1,2,3,...,n]$. Each round obfuscates its block matrix $C_{I_i}$ one by one until all blocks have been encrypted, which is also the end of each round. After the end of the round, use the new key to generate a brand new complete chaotic sequence for a new round of processing.

$\mathbf{Step ~4}$. Confusing the i-th block multiplexing chaotic matrix

After generating the previous ciphertext image $C_z$, extract its image feature values as the initial parameters of chaos, and generate a chaotic sequence with a length of $\frac{H}{n}\times W$. Next, it is reconstructed into a chaotic confusion matrix $K_i$ with height of $\frac{H}{n}$ and width of *W*, ensuring that its size is the same as the chaotic matrix $C_{I_i}$ after piecemeal. Finally, use $K_i$ and $C_{I_i}$ to perform the confusion operation (size $\frac{H}{n}\times W$), with other positions unchanged, to obtain a multiplexed chaotic matrix $R_i$ (size $H\times W$) for encrypting the plaintext image of the next frame, as follows:

13 $$\begin{aligned} R_i\left( \frac{H}{n}\times (z-1)+1:\frac{H}{n} \times z,1:W\right) =K_i \oplus C_{I_i} \end{aligned}$$

where *z* represents the number of frames where the image is located, $z=[1,2,3,...,z]$; $R_i$ represents the reused chaotic matrix after the i-th block has been confused.

$\mathbf{Step ~5}$. Encrypt the next image

After getting $R_i$, encrypt the plaintext image $P_{z+1}$ of the next frame in this round to obtain its corresponding ciphertext image $C_{z+1}$. The operation is the same as the permutation-diffusion-substitution in Step 2. But at this time, the chaotic matrix has been obfuscated,which means that the chaos matrix has been processed in a multiplexed. The specific encryption details will change from frame to frame with the same encryption operation, resulting in different encryption results for each frame. For a multiplexed chaotic matrix $R_0$ that has been partitioned by $r_i$, the $n+1$ frame image can be encrypted. Therefore, the following two situations will occur, and the specific discussion is as follows:

- Case 1. $$n \le z+1$$

When the number of video frames in this round is less than or equal to $n+1$, that the encryption of this round of video frames has not ended, only one chaotic initial matrix $R_0$ needs to be generated to complete the encryption operation of this round of video frames. At this point, simply repeat steps 4–5 until all video frames in this round are encrypted.

- Case 2. $$n > z+1$$

When the number of frames in the processed video is greater than $n+1$, that the previous round of encryption has ended and the previous round of chaotic sequence has been completely confused and reused, the hash feature value of the image in the last frame of the previous round of ciphertext is extracted, perform XOR operation is performed with $key_1$ to generate $key_2$ as the initial parameter of the complete chaotic sequence generated in the new round. Then, repeat steps 1–5 until all video frame images in the video are encrypted.

#### Decryption process

Decryption can be seen as the inverse process of encryption, and this section takes a video of size $H \times W$ with *z* frames as an example to briefly explain the decryption steps.

$\mathbf{Step ~1}$. Multiplexed Chaotic Sequence Generation and Preprocessing

After Eq. (7), $key_1$ is generated as the initial parameter of the chaotic sequence $S_0$, which is preprocessed according to Eq. (8) to obtain the sequence *Q* by Eq. (8a), the sequence *S* by Eq. (8b), and the sequence *I* by Eq. (8c).

$\mathbf{Step ~2}$. Decrypt the first frame

The decryption process is presented below, with the first frame $C_1$ serving as an illustrative example:

(1) Solving random order substitution

The anti-random order substitution algorithm corresponding to Eq. (12) performs decryption operations, with the specific algorithm as follows:

14 $$\begin{aligned} M_{I_{i,j},j} = {\left\{ \begin{array}{ll} (C_{1{I_{i,j}},j}-C_{1{I_{i,j-1}},j-1}-\lfloor 2^{32} \times S_{I_{i,j},j}\rfloor )mod256 &{} for \quad i=[1,W], j=[2,W]\\ (C_{1{I_{i,j},j}}-C_{1I_{i-1,W},W}-\lfloor 2^{32} \times S_{I_{i,j},j}\rfloor )mod256 &{} for\quad i=[1,W], j=1\\ (C_{1I_{i,j},j}-T_{I_{H,W},W}-\lfloor 2^{32} \times S_{I_{i,j},j}\rfloor )mod256 &{} for\quad i=1, j=1\\ \end{array}\right. } \end{aligned}$$

From the final ciphertext *C* to the intermediate ciphertext *M*.

(2) Solving row and column diffusion

The decryption method corresponding to the diffusion of rows and columns in the encryption algorithm is as follows:

15 $$\begin{aligned} D_i= & {} {\left\{ \begin{array}{ll} (M_W-M_{W-1}-\lfloor Q_W \times 2^{10}\rfloor )mod256 &{} if\quad j=W\\ (M_j-M_{j-1}-D_{j+1}+\lfloor Q_j \times 2^{10}\rfloor )mod256 &{} if\quad j=[W-1, 2]\\ (M_j-D_W+\lfloor Q_j \times 2^{10}\rfloor )mod256 &{} if\quad j=1\\ \end{array}\right. } \end{aligned}$$

16 $$\begin{aligned} B_j= & {} {\left\{ \begin{array}{ll} (D_T-D_{T-1}-\lfloor S_i \times 2^{10}\rfloor )mod256 &{} if\quad i=H\\ (D_i-D-_{i-1}-B_{i+1}-\lfloor S_i \times 2^{10}\rfloor )mod256 &{} if\quad i=[H-1,2]\\ (D_1-B_H-\lfloor S_i \times 2^{10}\rfloor )mod256 &{} if\quad i=1\\ \end{array}\right. } \end{aligned}$$

The permutation-only image *B* is obtained from the middle ciphertext image *M*.

3) Decryption block permutation

According to Eq. (9), four keys $a_1,a_2,a_3,a_4,$ can be obtained, and the rest of the decryption process is shown in Section Block permutation to obtain the recovered plain image *P*.

$\mathbf{Step ~3}$. Blocking of multiplexed chaos matrices

After constructing the sequence $S_0$ as a multiplexed chaotic matrix $R_0$ with height *H* and width *W*, the rest of the process is the same as Step 3 of Section Encryption process to obtain the blocking matrix $C_{I_i}$ , where $i=[1,2,3..., n]$.

$\mathbf{Step ~4}$. Confusion multiplexing chaotic matrix

In the same way as Step 4 in Section Encryption process, the hash value of the current ciphertext image is read and processed to obtain $K_i$, which is confused with ${C_I}_i$ to obtain the multiplexed chaos matrix $R_i$ for decrypting the next frame.

$\mathbf{Step ~5}$. Decrypt the next frame

After getting $R_i$, the next cipher image is decrypted, and the specific operation is the same as the decryption steps in Step 2. Similarly, for the n-chunked multiplexed chaos matrix, the $n+1$ frames can be decrypted. If the total number of video frames is less than or equal to $n+1$, the recovered video is obtained by repeating Step 4 to Step 5; if the total number of video frames is greater than $n+1$, the $C_{n+1}$ image characteristic value is read, and the new *key* is obtained as the initial value of chaos after a bitwise XOR operation with $key_1$ to generate the multiplexed chaos matrix for the next round of decryption, and Step 1 to Step 5 is repeated.

## Results and analysis

### Experimental platform

For the experimental platform, we used a PC host with MATLAB R2022a experimental software installed. The processor of the PC is AMD Ryzen$^\text {TM}$ 9 5950X CPU with 3.88GHz, the memory size is 64GB, the hard disk size is 8TB, and the operating system is Windows 10. USC-SIPI image database was used in the experimental data selection.

### Video encryption illustration

**Figure 8:**
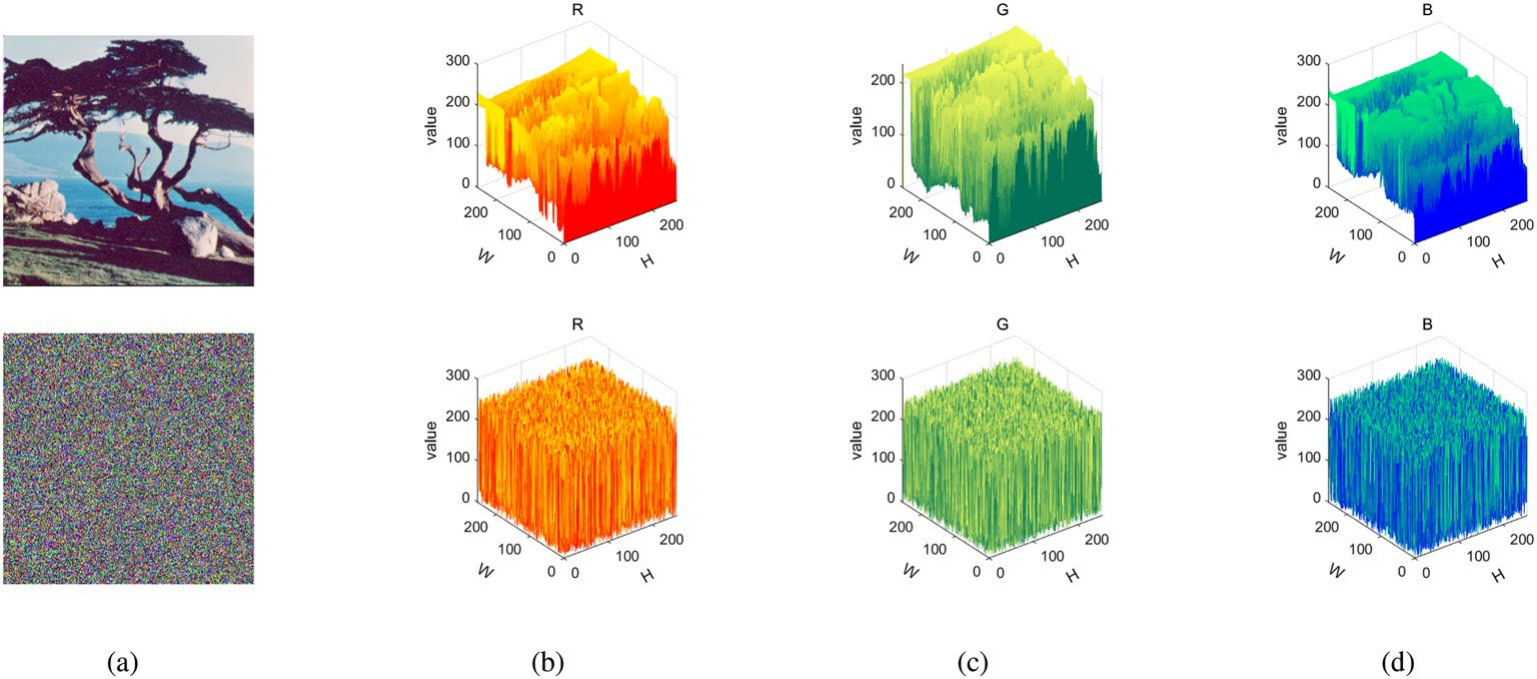
3D visualization of pixel distribution before and after encryption of plaintext and ciphertext RGB channels: (**a**) plain image and cipher image; (**b**) red channel; (**c**) green channel; (**d**) Blue channel.

**Figure 9:**
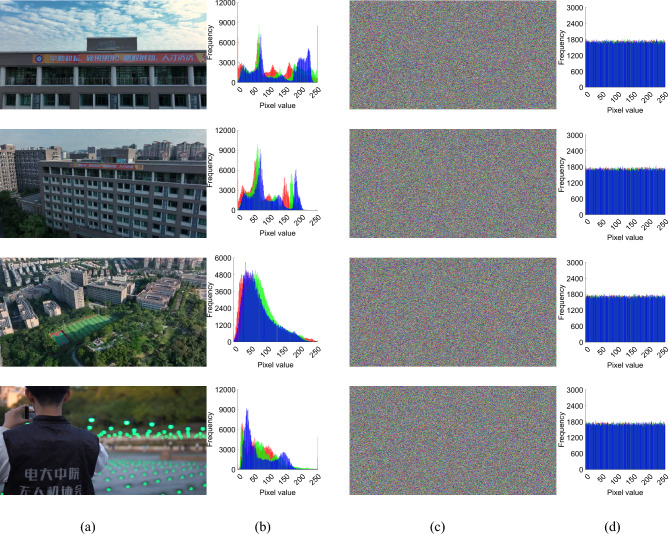
Encryption effect of different frame numbers of “Aerial School” video (**a**) original video images; (**b**) original video images histogram; (**c**) video frame images ciphertext; (**d**) video frame images ciphertext histogram.

**Figure 10:**
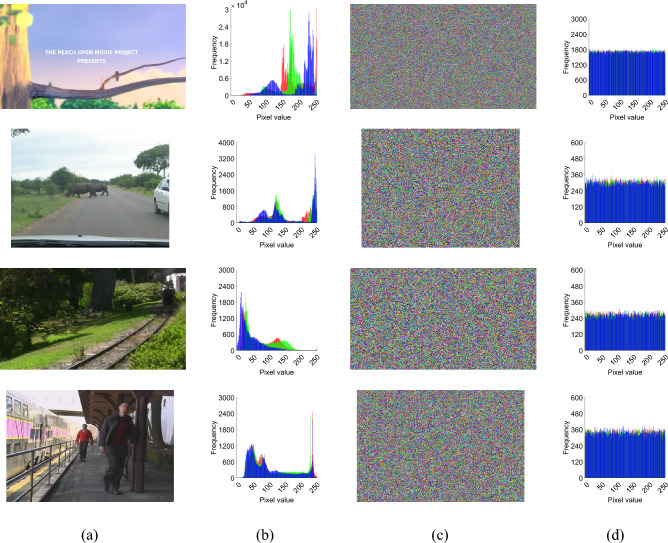
Different video encryption effects: (**a**) original video image; (**b**) original video image histogram; (**c**) video frame image ciphertext; (**d**) video frame image ciphertext histogram.

To illustrate the encryption effect, two experimental approaches were used. One is the same video we selected an arbitrary number of frames for illustration, and the other we selected different videos for illustration. They are Figs. 9 and 10, respectively. At the same time, we also used the “Tree” color image to test our encryption module, and its three-dimensional histogram results are shown in Fig. 8. From the experimental results show that the original video image presents a certain statistical pattern, while the histogram statistical characteristics of the encrypted video image present a noise-like distribution, which well hides the information of the image, thus improving the ability to resist statistical analysis attacks.

### Safety analysis

For video encryption, it is generally considered that a cryptosystem is secure when the cost of decrypting the ciphertext is greater than the cost of purchasing the video rights directly. The video encryption strength of the algorithm given in this paper is divided into three levels. One is chaos-based block permutation encryption of video frames, which provides basic protection of video data. The second one is the intra-frame pixel diffusion encryption, which further improves the security of the video. Finally, intra-frame pixel substitution protects the content of the video image more effectively. As can be seen in Figs. 9 and 10, the encryption effects of different videos and different frames in videos are compared. It is difficult to distinguish the differences, which proves that the algorithm is universal. In the whole encryption process, the multiplexing encryption of dynamic chaotic sequence greatly improves the efficiency of video encryption and decryption, and the improved four-dimensional chaotic system greatly improves the security of the algorithm.

### Statistical analysis

#### Histogram analysis

The histogram of the ciphertext is usually used to estimate how close the studied data sequence is to a uniform distribution. We calculated and plotted the histograms of the original video frames and the encrypted video frames. Figures 9b and 10b show the histograms of the original video frame images, and Figs. 9d and 10d show the histograms of the encrypted images of the original video frames. It can be seen that the histograms of the original video frame encrypted images are different from their corresponding histograms of the original video frames and are almost uniformly distributed.

#### Information entropy analysis

Entropy is the average amount of information obtained by observing the output of a source, and it refers to the degree of disorder in the system. The information entropy of an image is a measure of its randomness and unpredictability. For an information source *s*, the information entropy *H*(*s*) is defined as:

17 $$\begin{aligned} H(s)= \sum _{i=0}^M p(s_i) \log _2 \frac{1}{p(s_i)} \end{aligned}$$

where *M* is the number of symbols, $$s_i \in s$$, $$ p(s_i)$$ represents the probability of occurrence of symbol $$s_i$$. The entropy of a true random source is *H*(*s*), which corresponds to a uniform random information source. Ideally, the entropy of a cipher text should be 8 bits. However, the actual entropy is less than 8 bits. For evaluating cryptosystems, the entropy of a cryptosystem should ideally be close to the ideal value of 8 bits.

We calculate the information entropy of the original video frame and the corresponding video frame encrypted image, and give it in Tables 1 and 2. From Table 1, we find that the information entropy values of the proposed video frame encrypted image are 7.9999,7.9999,7.9998,7.9999, which are very close to the theoretical value of 8 bits. At the same time, in order not to lose generality, we also selected the other four videos, and the value of their information entropy is shown in Table 2. The information entropy values of their video frame encrypted images are 7.9998, 7.9992, 7.9989, 7.9994. In addition, we also calculate the value of the information entropy of the encryption module of the algorithm as shown in Table 3. At the same time, we use our encryption algorithm to compare with other classical encryption algorithms^40–44^. Our algorithm is closer to the theoretical value of 8, which is better than these classical encryption algorithms. This shows that the information leakage in the proposed encryption process is negligible.

**Table 1:** Information entropy of different frame numbers of “Aerial School” video.

Name	Aerial School			
Frame	147	169	284	905
plaintext	7.6434	7.8422	7.4517	7.3973
ciphertext	7.9999	7.9999	7.9998	7.9999

**Table 2:** Information entropy of different video images.

Name	Cartoon	Rhino	Train	Train Station
Frame	507	101	5	78
plaintext	7.2542	7.2689	7.2061	7.4908
ciphertext	7.9998	7.9992	7.9989	7.9994

**Table 3:** Information entropy values of the corresponding ciphertext image by different schemes.

		Proposed	Proposed	Ref.^40^	Ref.^41^	Ref.^42^	Ref.^43^	Ref.^44^
Images	Size	Plain image		Cipher image				
5.1.09	256 $\times$ 256	6.7093	7.9976	–	7.9971	7.9970	7.9973	7.9937
5.1.10	256 $\times$ 256	7.3118	7.9975	7.9973	7.9974	7.9970	7.9973	7.9975
5.1.11	256 $\times$ 256	6.4523	7.9971	–	7.9969	7.9974	7.9970	7.9970
5.1.12	256 $\times$ 256	6.7057	7.9962	–	7.9972	7.9972	7.9975	7.9972
5.1.13	256 $\times$ 256	1.5483	7.9967	7.9976	7.9969	7.9969	7.9972	7.9969
5.1.14	256 $\times$ 256	7.3424	7.9965	7.9974	7.9974	–	7.9970	7.9972
5.2.08	512 $\times$ 512	7.2010	7.9992	–	7.9993	–	7.9992	7.9992
5.2.09	512 $\times$ 512	6.9940	7.9993	–	7.9993	–	7.9994	7.9993
5.2.10	512 $\times$ 512	5.7056	7.9993	7.9992	7.9993	–	7.9993	7.9992
7.1.01	512 $\times$ 512	6.0274	7.9993	–	7.9991	–	7.9993	7.9992
7.1.02	512 $\times$ 512	4.0045	7.9993	7.9993	7.9992	–	7.9993	7.9932
7.1.03	512 $\times$ 512	5.4957	7.9992	7.9993	7.9993	–	7.9993	7.9993
7.1.04	512 $\times$ 512	6.1074	7.9993	7.9993	7.9993	–	7.9992	7.9993
7.1.05	512 $\times$ 512	6.5632	7.9994	7.9993	7.9992	–	7.9993	7.9992
7.1.06	512 $\times$ 512	6.6953	7.9993	7.9994	7.9993	–	7.9992	7.9993
7.1.07	512 $\times$ 512	5.9916	7.9994	7.9993	7.9993	–	7.9993	7.9993
7.1.08	512 $\times$ 512	5.0534	7.9993	7.9994	7.9993	–	7.9994	7.9991
7.1.09	512 $\times$ 512	6.1898	7.9993	7.9993	7.9992	–	7.9993	7.9992
7.1.10	512 $\times$ 512	5.9088	7.9994	7.9992	7.9993	–	7.9993	7.9991
boat.512	512 $\times$ 512	7.1914	7.9993	–	7.9994	–	7.9992	7.9992
ruler.512	512 $\times$ 512	0.5000	7.9994	7.9993	7.9992	–	7.9993	7.9993
5.3.01	1024 $\times$ 1024	7.5237	7.9998	7.9998	7.9998	–	7.9998	7.9998
5.3.02	1024 $\times$ 1024	6.8303	7.9998	7.9998	7.9998	–	7.9998	7.9998
7.2.01	1024 $\times$ 1024	5.6415	7.9998	7.9998	7.9998	–	7.9998	7.9998

#### Relevance analysis of video encryption

**Figure 11:**
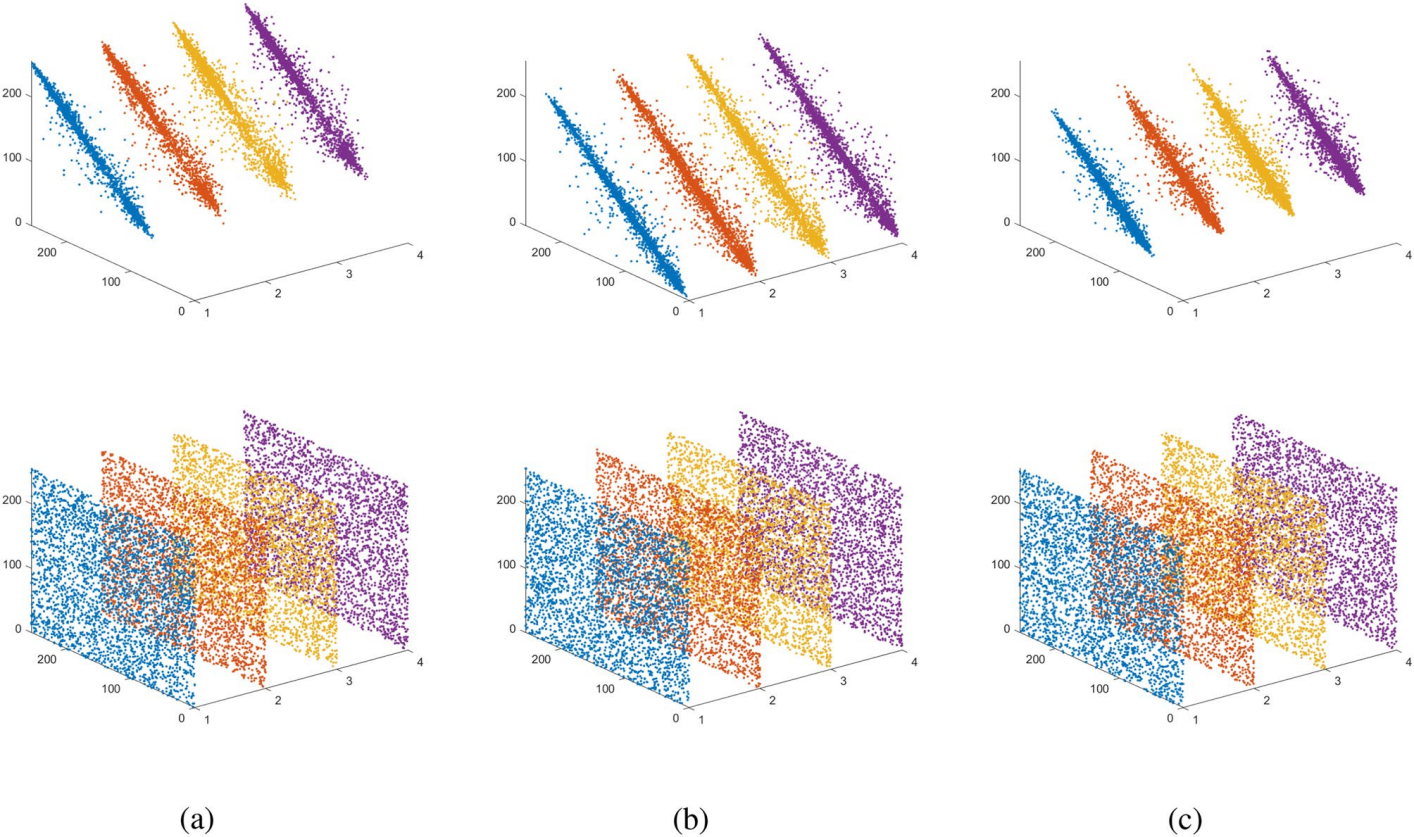
The correlation between Horizontal/Vertical/Diagonal/Anti-diagonal pixels of video plaintext and ciphertext images (**a**) Red channel; (**b**) Green channel; (**c**) Blue channel.

Correlation analysis allows judging the strengths and weaknesses of encryption algorithms in eliminating plaintext pixel correlation. For this purpose, 3000 pairs of adjacent pixel points are randomly selected from plaintext and ciphertext, and the correlation coefficients of adjacent pixels in horizontal, vertical, diagonal and anti-diagonal directions are calculated and their correlation scatter plots are drawn, as shown in Fig. 11. Table 4 shows the comparison between the proposed scheme and the classical encryption schemes in recent years. It is found that the adjacent pixel points of plaintext in horizontal, vertical, diagonal and anti-diagonal directions show a concentrated distribution with statistical characteristics, while the adjacent pixel points of ciphertext show a random distribution in horizontal, vertical, diagonal and anti-diagonal directions, which shows that the adjacent pixels of video images encrypted by the algorithm of this paper have almost no correlation, providing a strong guarantee for information security.

**Table 4:** Comparison results of correlation coefficients of adjacent pixels.

Component	Direction	Original Image	Proposed	Ref.^45^	Ref.^46^	Ref.^47^
R	Horizontal	0.9889	0.0036	$-$ 0.0063	0.0076	0.0023
	Vertical	0.9776	0.0088	$-$ 0.0016	0.0017	$-$ 0.0130
	Diagonal	0.9663	$-$ 0.0034	0.0156	0.0110	$-$ 0.0061
	Anti-diagonal	0.9804	$-$ 0.0255	–	–	–
G	Horizontal	0.9833	$-$ 0.0243	$-$ 0.0032	$-$ 0.0048	$-$ 0.0236
	Vertical	0.9664	$-$ 0.0068	0.0335	0.0274	0.0308
	Diagonal	0.9588	$-$ 0.0177	$-$ 0.0095	0.0342	$-$ 0.0179
	Anti-diagonal	0.9655	$-$ 0.0501	–	–	–
B	Horizontal	0.9578	0.0146	$-$ 0.0044	$-$0.0056	$-$ 0.0266
	Vertical	0.9265	$-$ 0.0106	$-$ 0.0079	0.0150	$-$ 0.0057
	Diagonal	0.9172	0.0054	0.0034	$-$ 0.0115	0.0378
	Anti-diagonal	0.9227	$-$ 0.0151	–	–	–

#### Differential statistics analysis

**Figure 12:**
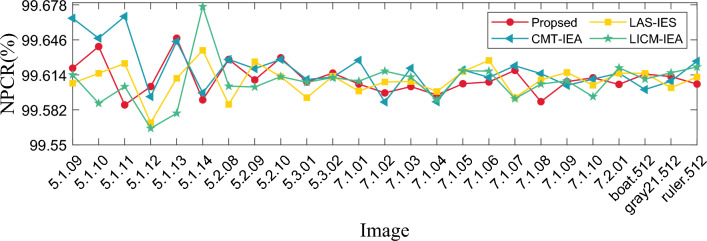
Comparison results of NPCR visualization with different algorithms.

**Figure 13:**
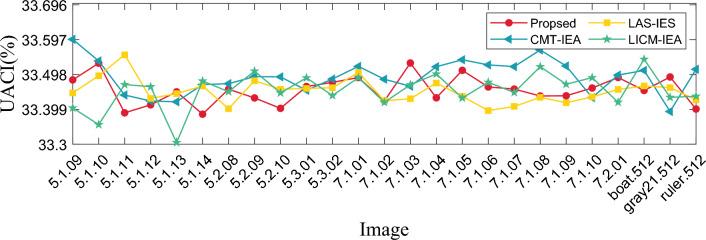
Comparison results of UACI visualization with different algorithms.

In the image encryption algorithm, the metric of sensitivity to plaintext usually uses Number of Pixels Change Rate (NPCR) and Unified Average Changing Intensity (UACI). In video encryption, the video is composed of multiple frames, we selected different frames in one video and random frames inside different videos to analyze, and the calculation equation is defined as:

18 $$\begin{aligned} {\left\{ \begin{array}{ll} \text{ NPCR }= \frac{1}{H\times W} \times \sum _{i=1}^H \sum _{j=1}^WD(i,j)\times 100\%, \\ \\ \text{ UACI }= \frac{1}{H\times W} \times \sum _{i=1}^H \sum _{j=1}^W\frac{|v_1(i,j)-v_2(i,j)|}{255}\times 100\%. \\ \end{array}\right. } \end{aligned}$$

where $H\times W$ is the size of the image; $v_1, v_2$ are the ciphertext before and after changing one pixel of the plaintext, respectively; *D* is as shown below:

19 $$\begin{aligned} D = {\left\{ \begin{array}{ll} 0,v_1(i,j)=v_2(i,j), \\ 1,v_1(i,j)\ne v_2(i,j).\\ \end{array}\right. } \end{aligned}$$

where $v_1(i,j)$ denotes the pixel value of the ciphertext pixel before it is changed. $v_2(i,j)$ denotes the pixel value of the ciphertext pixel after changing the pixel value at a point in the plaintext. The values of NPCR and UACI are calculated using Eq. (18), as shown in Tables 5 and 6. At the same time, in order not to lose generality, we also calculate the NPCR and UACI values of the encryption module of this algorithm as shown in Table 7. At the same time, we use our encryption algorithm to compare with other classical encryption algorithms as shown in Figs. 12 and 13. Our algorithm is closer to the theoretical value as shown in Table 8, which is superior to these classical encryption algorithms. Observing the above charts and data, it can be seen that the adjacent pixels of the plaintext have a strong correlation, while the adjacent pixels of the ciphertext have no obvious correlation. Therefore, it can be found from the experimental results that the encryption algorithm in this paper can effectively resist statistical analysis.

**Table 5:** NPCR and UACI values of different frame number images of “Aerial School” video frame.

Name	Frame	NPCR(%)	UACI(%)
Aerial School	147	99.5998	33.4993
	169	99.5986	33.4480
	284	99.6087	33.4775
	905	99.6158	33.4513

**Table 6:** NPCR and UACI values of different video frame.

Name	Frame	NPCR(%)	UACI(%)
Cartoon	507	99.6033	33.4161
Rhinoceros	101	99.5807	33.4244
Train	5	99.6404	33.4716
Train station	78	99.6262	33.4442

**Table 7:** NPCR and UACI values of different images.

Image	NPCR(%)				UACI(%)			
	R	G	B	Mean	R	G	B	Mean
4.1.01	99.6185	99.6429	99.5789	99.6134	33.4024	33.4509	33.4270	33.4268
4.1.02	99.6109	99.6033	99.5682	99.5941	33.6640	33.3655	33.4546	33.4947
4.1.03	99.6384	99.5987	99.5850	99.6074	33.3456	33.5458	33.5296	33.4737
4.1.04	99.5636	99.5972	99.6216	99.5941	33.5206	33.4802	33.3561	33.4523
4.1.05	99.5880	99.6109	99.6277	99.6089	33.2602	33.5222	33.5925	33.4583
4.1.06	99.6201	99.6078	99.6613	99.6297	33.5324	33.5704	33.5562	33.5530
4.1.07	99.5941	99.6033	99.6017	99.5997	33.5671	33.5412	33.5100	33.5394
4.1.08	99.5972	99.5728	99.5819	99.5840	33.4729	33.5646	33.4703	33.5026
4.2.01	99.5991	99.6113	99.5960	99.6021	33.4638	33.5410	33.4968	33.5005
4.2.03	99.6040	99.6025	99.6101	99.6055	33.4596	33.3668	33.4730	33.4331
4.2.05	99.5850	99.6170	99.6147	99.6056	33.4692	33.4080	33.4338	33.4370
4.2.06	99.6208	99.5872	99.6105	99.6062	33.4700	33.3770	33.3156	33.3875
4.2.07	99.6231	99.6174	99.5846	99.6084	33.4719	33.4436	33.4702	33.4503

**Table 8:** The average values of NPCR and UACI were compared with other algorithms.

	Proposed	CMT-IEA^48^	LAS-IES^49^	LICM-IEA^50^
NPCR	99.6103	99.6462	99.6092	99.6176
UACI	33.4748	33.5555	33.4359	33.4198

#### Video frame image quality analysis

Peak Signal to Noise Ratio (PSNR) and Structural Similarity (SSIM) are commonly used as a tool to weigh the quality of encryption in the image processing field. Mean Square Error (MSE) is a part of PSNR which is defined as:

20 $$\begin{aligned} {\left\{ \begin{array}{ll} \text{ MSE }=\frac{1}{H\times W}\sum _{i=1}^H \sum _{j=1}^W(X(i,j)-Y(i,j))^2\\ \text{ PSNR }=10\times \log _{10} \left( \frac{Q^2}{MSE} \right) \\ \end{array}\right. } \end{aligned}$$

where MSE denotes the mean square error of the plaintext image *X* and the ciphertext image *Y*. The height and width of the image are denoted by *H* and *W*, respectively. And *Q* denotes the pixel level of the image. SSIM is a measure of the similarity of two images, defined as:

21 $$\begin{aligned} \text{ SSIM }(X,Y)= \frac{(2\mu _X\mu _Y+(0.01L)^2)(2\sigma _{XY}+(0.03L)^2)}{(\mu _X^2+\mu _Y^2+(0.01L)^2)(\sigma _X^2+\sigma _Y^2+(0.03L)^2)} \end{aligned}$$

where $\mu _X$ and $\mu _Y$ denote the mean values of image *X* and *Y*, respectively, the standard deviation of image *X* and *Y*, respectively, and *L* denotes the dynamic range of pixel values. The values of PSNR and SSIM are calculated by using Eq. (20) and (21), as shown in Tablestest results are detailed in the Tables 9 and 10. At the same time, in order not to lose generality, we also selected many images to test our encryption module, and its results are shown in Table 11. The PSNR value of the encrypted image should be less than 10db, and the range of SSIM should be from -1 to 1. The closer the image is, the closer the absolute value of SSIM is to 1. Therefore, the value of SSIM should be floating up and down from 0 after encryption. The experimental results show that our encryption algorithm has a good encryption effect.

**Table 9:** PSNR, MSE and SSIM values of different frame numbers of “Aerial School” video.

Name	Aerial School		
Frame	PSNR(dB)	MSE	SSIM
147	7.6915	11065	0.0078
169	8.0924	10089	0.0085
284	8.1098	10049	0.0084
905	7.8019	10787	0.0084

**Table 10:** PSNR, MSE and SSIM values of different video images.

Name	Frame	PSNR	MSE	SSIM
Cartoon	507	7.6705	11118	0.0100
Rhinoceros	101	7.6508	11169	0.0093
Train	5	7.4531	11689	0.0086
Train Station	78	7.9578	10406	0.0102

**Table 11:** PSNR, MSE and SSIM values of different images.

Filename	Description	Size	Type	MSE	PSNR	SSIM
4.1.01	Female (NTSC test image)	256	Color	12114.00	7.2981	0.0077
4.1.02	Couple (NTSC test image)	256	Color	15344.00	6.2714	0.0040
4.1.03	Female (from Bell Labs?)	256	Color	6590.50	9.9417	0.0112
4.1.04	Female	256	Color	8521.80	8.8255	0.0100
4.1.05	House	256	Color	8362.10	8.9077	0.0105
4.1.06	Tree	256	Color	9939.60	8.1571	0.0090
4.1.07	Jelly beans	256	Color	8980.70	8.5977	0.0098
4.1.08	Jelly beans	256	Color	8840.20	8.6662	0.0110
4.2.01	Splash	512	Color	11203.00	7.6376	0.0090
4.2.03	Mandrill (a.k.a. Baboon)	512	Color	8612.10	8.7797	0.0104
4.2.05	Airplane(F-16)	512	Color	10367.00	7.9744	0.0098
4.2.06	Sailboat on lake	512	Color	10104.00	8.0858	0.0080
4.2.07	Peppers	512	Color	10109.00	8.0837	0.0089
5.1.09	Moon surface	256	Gray	6269.10	10.1588	0.0098
5.1.10	Aerial	256	Gray	7664.70	9.2858	0.0098
5.1.11	Airplane	256	Gray	10995.00	7.7187	0.0098
5.1.12	Clock	256	Gray	12133.00	7.2913	0.0107
5.1.13	Resolution chart	256	Gray	20876.00	4.9343	0.0064
5.1.14	Chemical plant	512	Gray	7762.00	9.2311	0.0091
5.2.08	Couple (NTSC test image)	512	Gray	7077.70	9.6319	0.0097
5.2.09	Aerial	512	Gray	9837.60	8.2019	0.0091
5.2.10	Stream and bridge	1024	Gray	8669.70	8.7508	0.0085
5.3.01	Male	1024	Gray	10298.00	8.0033	0.0086
5.3.02	Airport	512	Gray	8681.50	8.7449	0.0091
7.1.01	Truck	512	Gray	6616.70	9.9244	0.0106
7.1.02	Airplane	512	Gray	8225.90	8.9790	0.0110
7.1.03	Tank	512	Gray	6237.20	10.1809	0.0108
7.1.04	Car and APCs	512	Gray	6796.20	9.8081	0.0109
7.1.05	Truck and APCs	512	Gray	7118.80	9.6067	0.0106
7.1.06	Truck and aPCs	512	Gray	7952.50	9.1258	0.0095
7.1.07	Tank	512	Gray	6424.00	10.0528	0.0103
7.1.08	APC	512	Gray	6039.80	10.3206	0.0109
7.1.09	Tank	512	Gray	6751.60	9.8368	0.0107
7.1.10	Car and APCs	1024	Gray	6252.60	10.1702	0.0108
7.2.01	Airplane (U-2)	512	Gray	15168.00	6.3214	0.0046
boat.512	Fishing Boat	512	Gray	7638.20	9.3009	0.0086
gray21.512	21 level step wedge	512	Gray	11378.00	7.5703	0.0090
ruler.512	Pixel ruler	512	Gray	21702.00	4.7657	0.0075
Mean				9569.84	8.5564	0.0094

### Key space analysis

The key space refers to the set of all possible keys that can be used to generate a key, and the size of the key space depends on the length of the security key, which is one of the most important characteristics that determine the strength of a cryptosystem. The image encryption algorithm designed in this paper uses a 4D autonomous hyperchaotic system, whose key space can be expressed as $S\in \left\{ key_1,key_2,a_1,a_2,a_3,a_4,MD5 \right\}$, where $key_1,key_2$ are the key parameter with the precision of ${{10}^{-16}}$, $a_1,a_2,a_3,a_4$ are the key parameter with the precision of ${{2}^{-16}}$ and *MD*5 is the hash value introduced to enhance the key space, which can generate a 128 bit hash value. After calculation, the key space size of this encryption scheme is about ${{10}^{2\times 16}}\times {{2}^{16\times 4}}\times {{2}^{128}}\approx {{2}^{295}}$ and the key length reaches 295 bits in this paper. Usually, the larger the key space is, the more computational resources and time are required to break the encryption algorithm. Therefore, the key space generated by the encryption algorithm in this paper is large enough to resist any form of brute force attack.The key space comparison is shown in Table 12.

**Table 12:** Key space size comparison table.

This Article	Ref.^51^	Ref.^52^	Ref.^53^	Ref.^54^	Ref.^55^
295	128	166	154	224	256

### Sensitivity analysis

In this section, the performance metrics of the algorithm are analyzed in terms of both key and plaintext sensitivity, respectively. The security algorithm should be highly sensitive, which means that if there is a slight change in the key or plaintext image information during encryption or decryption, it will have a huge impact on the result of the subsequent encryption.

#### Analysis of sensitivity to the key

**Figure 14:**
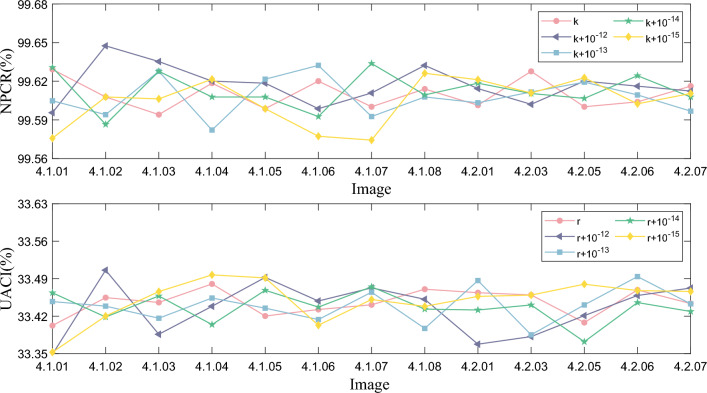
Comparison results of NPCR and UACI visualization with different disturbance values.

Key sensitivity is analyzed by analyzing the ciphertext obtained when encrypting the same image using two slightly different keys. In this section, we encrypt the plaintext image by using the original key, which defined as *key*, and the scrambling key, which defined as $key+{{10}^{-12}}, key+{{10}^{-13}}, key+{{10}^{-14}}, key+{{10}^{-15}}$, respectively. Then, compare the difference between the encrypted ciphertexts by calculating the NPCR and UACI. Where NPCR and UACI are defined as shown in Eq. (18). The results are shown in Fig. 14 and Table 13, and we can find that the difference between the two ciphertext images is very large when the scrambling is added to the key, and their NPCR and UACI values are very close to the ideal values of 99.6094$\%$ and 33.4635$\%$.

**Table 13:** Key sensitivity test results.

Value	0		$10^{-12}$		$10^{-13}$		$10^{-14}$		$10^{-15}$	
Index	NPCR	UACI	NPCR	UACI	NPCR	UACI	NPCR	UACI	NPCR	UACI
4.1.01	99.6292	33.4024	99.5956	33.3488	99.6048	33.4472	99.6307	33.4636	99.5758	33.3525
4.1.02	99.6078	33.4546	99.6475	33.5059	99.5941	33.4387	99.5865	33.4184	99.6078	33.4202
4.1.03	99.5941	33.3456	99.6353	33.3861	99.6277	33.4161	99.6277	33.5077	99.6063	33.4659
4.1.04	99.6185	33.4802	99.6201	33.4384	99.5621	33.4539	99.6078	33.4042	99.6216	33.5771
4.1.05	99.5987	33.3602	99.6185	33.4924	99.6216	33.4349	99.6078	33.4683	99.5987	33.4915
4.1.06	99.6201	33.3324	99.5987	33.4483	99.6323	33.4136	99.5926	33.5369	99.5773	33.4030
4.1.07	99.6002	33.4412	99.6109	33.4728	99.5926	33.4648	99.6338	33.5748	99.5743	33.4511
4.1.08	99.6140	33.4703	99.6323	33.4515	99.6078	33.3971	99.6094	33.4331	99.6262	33.4382
4.2.01	99.6014	33.4638	99.6143	33.3676	99.6033	33.4865	99.6185	33.4315	99.6212	33.4569
4.2.03	99.6277	33.4596	99.6021	33.3818	99.6117	33.3851	99.6105	33.5409	99.6109	33.4593
4.2.05	99.6002	33.4080	99.6201	33.4210	99.6193	33.4410	99.6067	33.3722	99.6227	33.4797
4.2.06	99.6040	33.4690	99.6162	33.4586	99.6094	33.4939	99.6243	33.3454	99.6025	33.4678
4.2.07	99.6162	33.4436	99.6124	33.4725	99.5968	33.4429	99.6078	33.5287	99.6105	33.4661

#### Analysis of plaintext sensitivity

**Figure 15:**
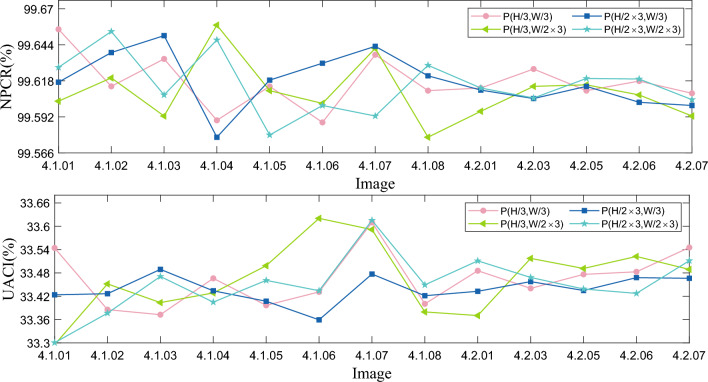
Comparison results of NPCR and UACI visualization with different locations.

The plaintext sensitivity is the degree of change in the corresponding ciphertext when changing the pixels of the plaintext. If the algorithm lacks plaintext sensitivity, an attacker is likely to decipher the algorithm by analyzing the difference between the plaintext and ciphertext pairs. Therefore, the algorithm’s plaintext sensitivity is the key to its resistance to plaintext attacks. In this section, we analyze the sensitivity of the proposed algorithm to the plaintext image by adding 1 to the pixel value of the plaintext image at (H/3, W/3), (H/3, 2$\times$W/3), (2$\times$H/3, W/3) and (2$\times$H/3, 2$\times$W/3), and the results can be obtained by comparing its NPCR and UACI values. The results are shown in Fig. 15 and Table 14. From the experimental results, it can be seen that the NPCR between the ciphertext and the original ciphertext is very close to the ideal value of 99.6094$\%$ and the UACI is also very close to the ideal value of 33.4635$\%$ when the change of the pixel values at the selected locations is 1. This indicates that the ciphertext image has changed significantly, making it impossible for an attacker to compromise the algorithm by comparing the differences between the ciphertexts, and therefore, the algorithm proposed in this paper is sufficient to resist plaintext attacks.

**Table 14:** Plaintext sensitivity test results.

Position	(H/3,W/3)		(H/3,2$\times$W/3)		(2$\times$H/3,W/3)		(2$\times$H/3,2$\times$W/3)	
Index	NPCR	UACI	NPCR	UACI	NPCR	UACI	NPCR	UACI
4.1.01	99.6552	33.5441	99.6033	33.2953	99.6170	33.4236	99.6277	33.3005
4.1.02	99.6140	33.3855	99.6201	33.4517	99.6384	33.4265	99.6536	33.3765
4.1.03	99.6338	33.3725	99.5926	33.4036	99.6506	33.4891	99.6078	33.4708
4.1.04	99.5895	33.4660	99.6582	33.4285	99.5773	33.4339	99.6475	33.4048
4.1.05	99.6140	33.3966	99.6109	33.4985	99.6185	33.4071	99.5789	33.4608
4.1.06	99.5880	33.4311	99.6017	33.6203	99.6307	33.3593	99.6002	33.4342
4.1.07	99.6368	33.6100	99.6414	33.5914	99.6429	33.4770	99.5926	33.6152
4.1.08	99.6109	33.4004	99.5773	33.3796	99.6216	33.4213	99.6292	33.4492
4.2.01	99.6128	33.4856	99.5960	33.3703	99.6113	33.4327	99.6128	33.5110
4.2.03	99.6265	33.4402	99.6140	33.5173	99.6052	33.4576	99.6056	33.4683
4.2.05	99.6109	33.4762	99.6151	33.4914	99.6140	33.4347	99.6197	33.4386
4.2.06	99.6178	33.4828	99.6078	33.5223	99.6025	33.4678	99.6193	33.4273
4.2.07	99.6090	33.5454	99.5926	33.4890	99.6002	33.4661	99.6044	33.5114

### Efficiency analysis

#### Time analysis

The time spent in the encryption and decryption process of the encryption scheme is also a key parameter to evaluate the performance of the scheme. The detailed parameters of the different types of video sources used in this article are shown in Table 15. At the same time, this paper analyzes the time of the proposed video encryption algorithm. Table 16 shows the time required for the proposed encryption method to encrypt each frame and the time required for the proposed encryption method to decrypt each frame of image ciphertext.

**Table 15:** Properties of Sample videos.

Video Name	Frame Dimensions	Data rate(Kbps)	Frame rate(Frames persecond)	Total Number of Frames
Aerial School	2406 $\times$ 1267	8192	24	2400
Cartoon	640$\times$360	678	24	603
Train	352$\times$192	529	25	261
Flamingo	352$\times$192	308	25	327
Rhino	320$\times$240	496	25	113
Train Station	360$\times$240	400	30	600

**Table 16:** Encryption and Decryption time of sample videos (Unit : seconds).

	Train	Flamingo	Rhino	Aerial school	Cartoon
Encryption time	0.2317	0.2351	0.2807	7.7743	0.8304
Decryption time	0.2989	0.3089	0.3605	9.9861	0.9751

#### Analysis of multiplexing sequence effect

In order to show the advantages of the multiplexing sequence algorithm proposed in this paper more clearly, we selected the multiplexing times of 0,2,4,8 and 16 to analyze the encryption time of the test video. The results are shown in Table 17. The algorithm of multiplexing sequence can significantly reduce the encryption time of the algorithm, which can provide a new idea for the subsequent chaotic video encryption algorithm.

**Table 17:** Encryption time results under different multiplexing sequence frequencies (Unit : seconds).

	0	2	4	8	16
Train	75.726756	67.272096	60.4737	56.581978	53.608762
Flamingo	93.2138	82.142452	76.8777	67.62449	64.443909
Rhino	40.96111	35.558809	31.7191	29.17811	27.683762
Cartoon	624.592186	554.303692	500.7312	465.336402	444.75226

### Chaotic sequence test

#### NIST test

NIST test refers to a series of tests and evaluations conducted by the National Institute of Standards and Technology in the United States, aimed at measuring and evaluating the performance, safety, and compliance with standards of various technologies and systems. NIST has established Test suite, benchmarks, and guidelines for the security verification of software, hardware, and other related technologies. These NIST tests are widely used globally, especially in the field of information security.

The NIST Encryption Test suite is used to evaluate the strength and security of encryption algorithms. In order to evaluate the randomness of chaotic sequences, this article used NIST 800-22 components for reliability testing of chaotic sequences. NIST Test suite is a statistical software package that contains 16 tests designed to test binary sequences of any length generated by hardware or software based cryptographic random or pseudorandom generators. The test results are detailed in the Table 18. Through these results, we can verify the effectiveness of the expected digital image encryption tool in NIST testing, and also indicate that the chaotic sequence required for encryption has successfully passed the test.

**Table 18:** NIST test results.

Statistical tests	$P-value$	Results
Frequency (Monobit) test	0.350485	successful
Block-frequency test	0.350485	successful
Cumulative-Sums test	0.350485	successful
Runs test	0.534146	successful
Longest-run test	0.017912	successful
Binary matrix rank test	0.739918	successful
Discrete Fourier transform test	0.739918	successful
Non-overlapping templates test	0.000199	successful
Overlapping templates test	0.350485	successful
Maurer’s Universal statistical test	0.911413	successful
Approximate Entropy Test	0.739918	successful
Random-excursions test (x = −4)	0.022503	successful
Random-excursions variant test (x = −9)	0.022503	successful
Serial test-1	0.739918	successful
Serial test-2	0.911413	successful
Linear-complexity test	0.350485	successful

#### The 0-1 Gottwald Melbourne test

**Figure 16:**
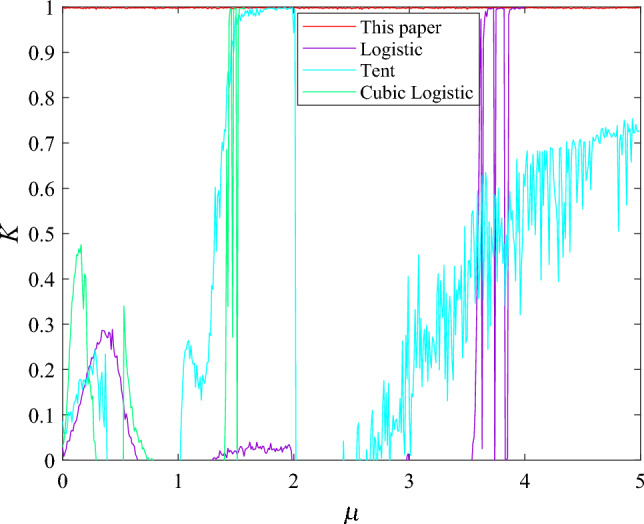
0-1 Gottwald Melbourne test result.

The 0-1 Gottwald Melbourne test can determine regular and chaotic motions by calculating the parameter *K* asymptotically close to 0 or 1. In the 0-1 Gottwald Melbourne test, the average result of 10,000 times has a *K* value of 0.9977, which is close to the theoretical value of 1, and verifies the excellent performance of the chaotic system. The results are shown in Fig. 16.

## Conclusion

This paper proposes a new video secure transmission algorithm based on 4D autonomous hyperchaotic system. The algorithm uses block permutation and dynamic multiplexing sequence encryption algorithm to enhance security. Specifically, the algorithm uses block permutation encryption and diffusion obfuscation based on dynamically multiplexed chaotic sequences, and considers the ciphertext information of each frame to process or generate dynamically multiplexed sequences. Compared with the traditional chaotic generation, the reuse sequence of this algorithm is more efficient. By using this algorithm, it can be ensured that each frame of video is effectively encrypted, which increases the difficulty of estimation or recognition. Experiments show that the algorithm has high security and efficiency in resisting various cryptographic attacks. Therefore, the video encryption scheme proposed in this paper is of great significance to the designers of chaotic encryption schemes, which can provide reference for them and improve the security and reliability of video encryption technology. In summary, this method is expected to provide new design ideas for video transmission security encryption under the background of big data era, and better improve the security and efficiency of video information in the transmission process.

## Data Availability

The datasets used and analysed during the current study available from the corresponding author on reasonable request. All data generated or analysed during this study are included in this published article.
